# Effect of Physical Activity/Exercise on Cardiorespiratory Fitness in Children and Adolescents with Type 1 Diabetes: A Scoping Review

**DOI:** 10.3390/ijerph20021407

**Published:** 2023-01-12

**Authors:** Xinyi Chang, Ziheng Wang, Hongzhi Guo, Yinghan Xu, Atsushi Ogihara

**Affiliations:** 1 Graduate School of Human Sciences, Waseda University, Tokorozawa 359-1192, Japan; 2 Graduate School of Biomedical Engineering, Tohoku University, Sendai 980-8579, Japan; 3 Faculty of Human Sciences, Waseda University, Tokorozawa 359-1192, Japan

**Keywords:** physical activity, exercise, type 1 diabetes, cardiorespiratory fitness, children

## Abstract

The most common type of diabetes among children and adolescents is type 1 diabetes mellitus (T1DM), which is associated with an increased risk of cardiovascular disease (CVD). Additionally, lower levels of cardiorespiratory fitness (CRF) are linked to an increased risk of CVD. Regular exercise is associated with a decreased risk of CVD and improved CRF. We conducted this scoping review to assess the effects of exercise on CRF in youth with T1DM. Three electronic databases (PubMed, Embase, and Cochrane Central Register of Controlled Trials) were used to search for the relevant literature. In this analysis, the PICOS method was used to select studies and was performed according to the Preferred Reporting Items for Systematic Reviews and Meta-Analyses Guidelines scoping review guidelines for the evaluation of the effects of physical activity and cardiac function; the criteria may include the type and intensity of physical activity, the duration of the intervention, peak oxygen consumption (VO_2_), peak minute ventilation (VE), and peak heart rate of cardiorespiratory fitness. Screening resulted in 434 records. Of these, nine articles were included in our study. These nine studies were experimental (noncontrolled trials or randomized controlled trials) (*n* = 7) and observational (cross-sectional) (*n* = 2), and could be used to evaluate the effectiveness of physical activity interventions on cardiac function. The effects of exercise on CRF in youth with T1DM vary according to the type, frequency, and intensity of the exercise. According to our review, the duration of exercise included in the studies did not meet the recommendations of the guidelines for youth with T1DM. Additionally, half of the studies revealed that exercise could optimize the lipid profile in youth with T1DM. Hence, this research is to provide an overview of the effects of physical activity and exercise on CRF, cardiovascular fitness, lipid profile, and blood pressure in youth with T1DM, as well as identified potential limitations of the existing studies. Nevertheless, the limited number of clinical studies employing exercise interventions for children and adolescents with T1DM emphasize the need for more studies in this area, and more specific modes of exercise should be developed in the future.

## 1. Introduction

Diabetes mellitus is the most prevalent chronic illness in the world [1]. Type 1 diabetes mellitus (T1DM) is the most common type of diabetes in children and adolescents worldwide. It is caused by the destruction of pancreatic β-cells, which produce insulin [2]. During 2002–2012, the overall prevalence of diagnosed T1DM in the United States was almost 25/100,000 and increased by 3% every year [3]. T1DM is associated with a high risk of cardiovascular disease (CVD) [4]. According to statistics, the prevalence of severe cardiovascular outcomes among people who develop T1DM at a young age is up to 30 times greater than that of healthy individuals, and children with T1DM are at a higher risk of developing CVD than healthy children [5]. Alternative prevention and treatment strategies are needed for the high prevalence of CVD in people with T1DM, and only a few pharmaceutical treatments are available [6].

Study has shown that lower levels of cardiorespiratory fitness (CRF) are associated with an increased risk of CVD [7]. Increasing physical activity and exercise among people with can help improve CRF [8]. Therefore, physical activities and exercise as a new strategy for prevention and treatment of T1DM patients.

Physical activity includes all movement that increases energy use, whereas exercise is planned, structured physical activity and exercise enhances cardiometabolic, bone, and muscle health [9]. Exercise programs and periodic physical activity are recommended as cornerstones of T1DM [10]. For children and adolescents diagnosed with diabetes, a minimum of 60 min per day of moderate-or-vigorous aerobic activity, muscle-strengthening, and bone-strengthening activities at least 3 days a week for patients with diabetes is recommended [6,11]. Previous studies have revealed that regular exercise is related to a decreased risk of mortality and CVD in patients with T1DM [6,12,13,14].

Although there is evidence that physical activity is related to decreased CVD in patients with T1DM, it is unclear whether exercise may help improve CRF in children and adolescents with T1DM. Therefore, a scoping review was conducted to evaluate the effects of physical activity on CRF in children and adolescents with T1DM. The findings of this scoping review may help develop suitable exercises for patients with T1DM.

## 2. Materials and Methods

This scoping review was conducted according to the (Table S1) Preferred Reporting Items for Systematic Reviews and Meta-Analyses scoping review guidelines (PRISMA-ScR) [15].

### 2.1. Information Sources and Searching Strategy

The search was performed systematically and independently by three investigators, using PubMed, Cochrane, and Embase. The search terms and combinations from the PICOS (population, intervention, comparison, outcome, study design) method associated with Boolean operators, including AND and OR, were used to refine the search. The search terms included: (“Adolescent*” OR “Child*” OR “Teen*” OR “Pre-Pubescent*” OR “Pediatric*” OR “Early Child*” OR “Paediatric*” OR “Toddler*” OR “Infant*” OR “Juvenile*” OR “Bab*” OR “Young Adult*”) AND (“Exercise*” OR “Resistance Training*” OR “Exercise Therapy*” OR “Motor Activity” OR “Aerobic*” OR “Training” OR “Physical Activity*” OR “Exertion” OR “Physical Fitness” OR “Yoga” OR “Tai Ji” OR “Dance Therapy*” OR “Pilates”) AND (“Type 1 Diabetes Mellitus*” OR “T1D” OR “Type 1 diabetes*”) AND (“Cardiorespiratory* Function” OR “Cardiorespiratory risk Factors” OR “Cardiovascular* Function” OR “Cardiovascular risk Factors”)

### 2.2. Inclusion Criteria

According to the report of the Surgeon General, “Physical Activity and Health”, Physical activity refers to any bodily movement that requires energy expenditure, and exercise is a specific type of physical activity that involves planned and repetitive movements [16]. For our study, the terms “physical activity” and “exercise” are interchangeable.

For this study, the PICOS method was used to select studies that met the following criteria: (1) population: patients aged 3–18 years old with a diagnosis of type 1 diabetes mellitus; (2) intervention: assessment of physical activity; (3) outcomes were glycosylated hemoglobin (HbA1) level; fasting blood glucose (FBG) level; peak oxygen consumption (VO_2_); peak minute ventilation (VE); peak heart rate; levels of triglycerides, total cholesterol, cholesterol, low-density lipoprotein (LDL) cholesterol, and high-density lipoprotein (HDL) cholesterol; systolic blood pressure (SBP); and diastolic blood pressure (DBP); and (4) study design: cross-sectional studies, pre–post studies, or randomized controlled trials (RCTs) published in English.

### 2.3. Study Selection

All search records were downloaded from three databases (PubMed, Embase, and the Cochrane Central Register of Controlled Trials) until August 2022. Duplicates were subsequently deleted using EndNote software. Three researchers reviewed all the titles and abstracts according to the inclusion criteria. Reasons for the exclusion of studies were provided. Any controversies were discussed by three researchers. 

### 2.4. Data Extraction and Synthesis

To fulfill the search requirements, data were extracted by three independent researchers. The data were as follows: (1) author, (2) publication year, (3) country, (4) study design, (5) experimental and control group details, (6) duration of exercise, and (7) key outcomes of CRF, cardiovascular fitness, lipid profile, and blood pressure and HbA1 level. All authors confirmed the final data.

## 3. Results

### 3.1. The Selection of Studies 

**Figure 1:**
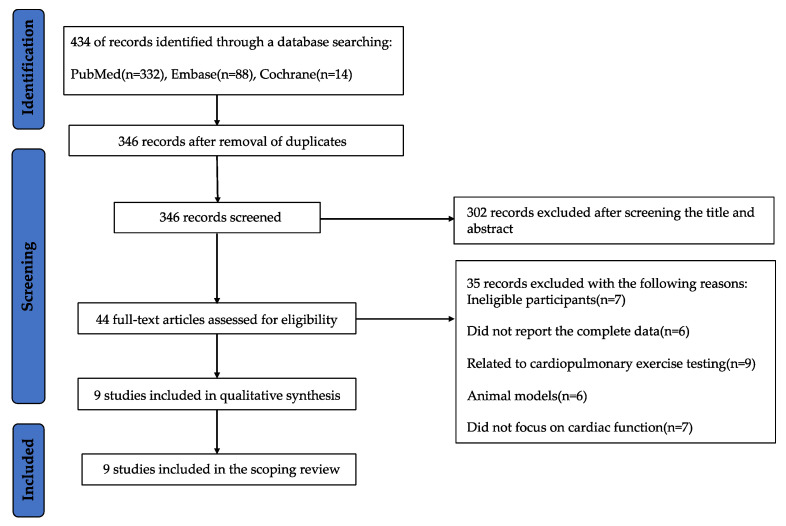
PRISMA flowchart of the screening of records.

The researchers identified 434 records from three databases: PubMed, Cochrane Central Register of Controlled Trials, and Embase. All records were loaded into EndNote software, and 88 duplicates were removed, leaving 346 articles. Three researchers selected the records by title and abstract, according to the inclusion criteria. Subsequently, the full text of 44 articles was evaluated for eligibility, and the reason for exclusion of each article was provided. Researchers identified that seven of the reports included ineligible participants, six did not report the complete data, nine reports were related to cardiopulmonary fitness testing, six reported animal models, and seven experiments did not focus on cardiac function. Thus, nine reports were included in the review from the database search. These studies were experimental (i.e., randomized or non-controlled trials) (*n* = 7) or observational (i.e., cohort, case-control, or cross-sectional) (*n* = 2). The PRISMA flowchart of the screened records is shown in Figure 1.

### 3.2. Characteristics of the Final Included Studies

The studies were published online from 1984 to 2021. The studies were performed in the United States (*n* = 3), Germany (*n* = 1), the Netherlands (*n* = 1), France (*n* = 1), Egypt (*n* = 1), Republic of Korea (*n* = 1), and Canada (*n* = 1). In addition, the studies included RCT (*n* = 4), pre–post studies (*n* = 3), and cross-sectional studies (*n* = 2). The characteristics of the selected studies are presented in Table 1. 

### 3.3. Characteristics of Study Participants

The number of participants involved in the groups in the studies ranged between 9 and 10,392 and the ages of these participants ranged from to 3–18 years. All the studies focused on children and teenagers diagnosed with T1DM. Seven studies included both boys and girls, except for Heyman et al., 2007 [21] and Shin et al., 2014 [23], whose studies included girls and boys, respectively. The characteristics of participants are presented in Table 2.

### 3.4. Characteristics of Exercise in the Included Studies

The duration of exercise in the included studies varied from 12 to 24 h per week. The number of exercise sessions per week ranged from to 2–5 per week. According to the type of training program, three studies included aerobic exercises or combined it with other exercises (strength or balance) [21,22,25], one study included regular exercise [17], one study included MVPA [18], one study included football sessions [20], and one study used interval walking training [23]. The remaining two were cross-sectional studies that evaluated the daily activities of the participants [19,24]. The relevant characteristics of the exercise interventions for each of the included studies are summarized in Table 1. 

These studies included a variety of techniques to identify heart function in adolescents with T1DM. Measurements included a treadmill running test [17], cycle ergometer [18,25], and metabolic gas analyzer to evaluate peak VO2, VE, and heart rate. Other measurements included laboratory methods for quantitative measurement of whole blood to detect HbA1 level and lipid profile [18], and FBG level was detected using a glucometer [20]. SBP and DBP were measured in mmHg using a mercury sphygmomanometer [20].

### 3.5. Pivotal Discoveries of the Included Studies Relating to Cardiac Function and Lipid Profile

For the purposes of this scoping review, the records were classified as follows: (a) physical activity and CRF; (b) physical activity and lipid profile; and (c) physical activity, blood sugar, and blood pressure. The key findings of CRF and lipid profile are presented in Table 3 and Table 4.

#### 3.5.1. CRF

Four articles evaluated the impact of exercise on CRF [17,18,23,25]. Faulkner 2010 et al., [18] and Michaliszyn 2010 et al., [25] reported that exercise sessions could significantly improve CRF. However, there was no significant improvement in peak VO_2_ or VE between the pre-and post-intervention groups in the two studies [17,23]. Only one study assessed the peak heart rate of participants after exercise sessions and found no significant improvement after the intervention. Table 3 summarizes the findings related to CRF.

#### 3.5.2. Lipid Profile

Seven studies explored the effect of exercise on lipid profile levels. A summary of the findings is presented in Table 4. Herbst 2007 et al., [25] and Mohammed et al., [24] carried out cross-sectional studies, and these two studies found that active exercises are associated with improvements in the levels of triglycerides, cholesterol, LDL cholesterol, and HDL cholesterol. Mohammed et al., [20] and Heyman 2007 et al., [21] reported that there was no significant change in the lipid profile of participants who attended an exercise program. The remaining three studies showed that some indicators of lipid profiles were significantly improved in patients with T1DM after taking part in exercise programs. 

#### 3.5.3. Blood Pressure and HbA1

Eight studies also explored the effect of physical activity on HbA1 levels, and two studies measured FBG levels. Among them, five studies found that HbA1 level was significantly decreased, and one study indicated that FBG levels declined, in the experimental groups. Shin 2014 et al., [23] found that in patients with T1DM, SBP and DBP significantly improved after an exercise intervention. Two studies revealed no significant improvement in SBP and DBP, indicating no association between physical activity and blood pressure. Additionally, Herbst 2007 et al., [19] and Mohammed 2014 et al., [24] observed marked improvements in DBP and SBP after patients attended exercise programs. 

In addition, Shin 2014 et al., [23] found that cardiac autonomic nervous system (ANS) activities related to the indicators, total power, low-frequency power, and very low-frequency power, improved significantly, and the Fourier pulsatility index (PI) and forward blood flow velocity did not change after exercise intervention.

## 4. Discussion

This is the first scoping review of the impact of exercise on CRF in children and adolescents with T1DM. The review identified nine published studies (including two cross-sectional studies) that satisfied the inclusion criteria. Based on these studies, exercise programs and their impacts on CRF vary. Aerobic exercise interventions included aerobics, a combination of aerobic and other training methods, regular exercise, walking, or football sessions. Half of the studies (5/9, 55.56%) did not measure CRF. Nine studies reported the lipid profile and blood pressure of participants, and several studies indicated that exercise may be related to improvements in blood pressure and lipid profile in patients with T1DM.

The first objective was to identify the types and durations of exercise approaches used in patients with T1DM to decrease cardiorespiratory dysfunction. As many exercise guidelines for T1DM exist, a comparison with the reviewed studies would be worthwhile. It is recommended that children with T1DM and healthy children receive 60 min of physical activity a day [26]. They are also recommended to perform high-intensity aerobic activities and activities that strengthen bones and muscles for at least three days a week [27,28]. However, according to our review, the exercise duration in the included studies varied from 12 to 24 weeks. The number of training sessions per week ranged from two to five per week, which did not meet the recommendations for the guidelines. 

The second objective of this study was to provide an overview of the effects of physical activity and exercise on CRF, cardiovascular fitness, lipid profile, blood pressure, and HbA1 levels in children and adolescents with T1DM. Only four studies reported peak VO_2_ or VE between the pre-and post-intervention groups, and the results varied widely. Therefore, CRF should be tested in more intervention groups. Lipids, lipoprotein(a), and apolipoproteins have been identified as potential cardiovascular risk factors. Lipoprotein(a) is a potential biomarker for atherosclerosis in patients with T1DM [29]. Half of the studies revealed that exercise could improve the lipid profile of patients with T1DM. Nevertheless, the limited number of clinical studies employing exercise interventions in children and adolescents with T1DM highlight the need for more research in this area. Only one study showed that the apolipoprotein B: apolipoprotein A-1 ratio significantly decreased in the experimental group, which raises the possibility of a possible role of protection on cardiovascular risks in adolescents with T1DM, as already observed in adults with T1DM [30]. 

The drawbacks of this scoping review are that two of the nine studies were cross-sectional and evaluated the relationship between physical activity/exercise and outcomes of CRF in adolescents with T1DM. More experimental studies, such as randomized controlled trials, are needed to verify these relationships. And limitation of this scoping review is the lack of measurement methods regarding cardiovascular fitness, such as flow-mediated dilation or brachial artery intima–media thickness [31,32]. Thus, no standardized measurements have been used to evaluate exercise interventions for CVD prevention. In addition, the relevant studies involved a wide variety of participants and were conducted in different countries. These patients may have received different therapies. As a result, comparing the studies was challenging, taking these limitations into account. Furthermore, only four studies provided measurements of VO_2_ restricts the ability to develop a comprehensive view of the impact on exercise duration. As such, further research should focus on including additional measures of exercise duration in order to provide a more comprehensive evaluation of the effects of physical activity and exercise training.

## 5. Conclusions

The effects of exercise on CRF in children and adolescents with T1DM vary with intensity, frequency, and type of exercise. Half of the studies revealed that exercise could improve the lipid profile in patients with T1DM. However, exercise interventions for children and adolescents with T1DM have been used in only a limited number of clinical studies, emphasizing the need for more research in this area and the development of more specific modes of exercise in the future.

## Figures and Tables

**Table 1:** The characteristics of the final included studies.

Study ID	Country	Study Design	Duration	Type, Intensity, Time, Frequency, and Grouping
Campaigne 1984 [17]	United States	Experimental (RCT)	12 weeks	Type and intensity: participated in the exercise sessions (running, games, and movement to music). Time and frequency: three 30 min sessions per week Grouping: IG: participated in the exercise sessions; CG: usual care.
Faulkner 2010 [18]	United States	Experimental (pre–post)	16 weeks	Type and intensity: aerobic exercises (between 60–75% peak heart rate, walking, kickboxing, dance revolution, etc.). Time and frequency: 60 min of moderate-to-vigorous physical activity (MVPA) each day for a goal of five days per week.
Herbst 2007 [19]	Germany	Observational (cross-sectional)		Grouping: regular physical activity (RPA) 0: none; RPA1: one–two times per week; RPA2: three times per week.
Mohammed 2021 [20]	Netherlands	Experimental (RCT)	12 weeks	Type and intensity: football program (between 79% and 84.6% peak heart rate). Time and frequency: two 90 min football exercises per week Grouping: football and diet (performed at high-intensity); football (performed at high-intensity); diet; the control group.
Heyman 2007 [21]	France	Experimental (RCT)	6 months	Type and intensity: exercises that combine aerobics and strength (running, aerobic dance, step, football, basketball, volleyball, rock climbing, gymnastics, etc.). Time and frequency: supervised session of two hours and unsupervised session for one hour per week. Grouping: training group: supervised session of two hours and unsupervised session for one hour per week; exercise about aerobics and strength; non-training control; usual care.
Salem 2010 [22]	Egypt	Experimental (RCT)	6 months	Type and intensity: Aerobic exercises (30 min, between 85–95% peak heart rate, e.g., cycling and treadmill). Leg extension and leg curl (30 min). Free strength and endurance exercises (10 min, e.g., bent calf knee raises, standing calf lifts, and toe curls). Flexibility exercises (5 min). Neuromuscular exercises (5 min). Warm-ups neck flexion (10 min, rotation, eversion, and foot inversion etc.). Grouping: Group A: participants did not attend the exercise program; Group B: participants joined the exercise sessions one times per week; Group C: participants attended the exercise sessions three times per week. Group B and C consisted of a balanced exercise regimen and an exercise training program (aerobic and anaerobic, different free strength and endurance, leg extension and leg curl, flexibility, and neuromuscular exercises).
Shin 2014 [23]	Republic of Korea	Experimental (pre–post)	12 weeks	Type and intensity: walking program (following a warm-up exercise at 4.0 km/h for 3 min, the exercise load was increased by increasing the incline grade by 2% every 2 min, at a walking speed of 4.8 km/h) Time and frequency: three times per week
Mohammed 2014 [24]	Canada	Observational (cross-sectional)		Grouping: very active (jumping, skating, running, skipping, swimming, cycling, and games that require significant movement); somewhat active (shopping, walking, or engaging in light household chores); somewhat inactive (reading, sitting, playing video games, watching television, time in front of the computer, activities that are performed mostly sitting down and playing games); inactive (resting, lying down, sleeping)
Michaliszyn 2010 [25]	United States	Experimental (pre–post)	16 weeks	Type and intensity: aerobic exercises (between 60–75% peak heart rate; walking, kickboxing, dance revolution, etc.) Time and frequency: no more than 60 min per day, five days per week Grouping: sedentary (<2.0 METS); light (2.0–3.0 METs); moderate (3.0–5.99 METs); moderate vigorous (≥3.0 metabolic equivalent (MET) units); vigorous (≥6.0 METs)

**Table 2:** The characteristics of participants.

Study ID	Grouping	Number (*n*)	Age (Range)	Age (Mean ± SD)	Males (*n*/%)
Campaigne 1984 [17]	IG	9	5–11	9.0 ± 0.5	N/A
	CG	10		8.5 ± 0.6	N/A
Faulkner 2010 [18]	T1DM	12	12–19	14.2 ± 1.4	9/75%
Herbst 2007 [19]	RPA0	10,392	3–18	12.7 ± 4.3	N/A
	RPA1	8607		12.6 ± 3.7	N/A
	RPA2	4252		13.9 ± 3.1	N/A
Mohammed 2021 [20]	CG	10	12–18	14.4 ± 2.0	N/A
	DG	10		15.6 ± 1.8	N/A
	FG	10		17.8 ± 0.4	N/A
	FDG	10		14.5 ± 1.4	N/A
Heyman 2007 [21]	Training Group	9	<18.5	15.9 ± 1.5	0/0
	Non-training Control	10		16.3 ± 1.2	0/0
Salem 2010 [22]	Group A	48	12–18	15 ± 2.4	N/A
	Group B	75		14.7 ± 2.2	N/A
	Group C	73		14.5 ± 2.4	N/A
Shin 2014 [23]	T1DM	15	13 ± 1	13.0 ± 1.0	15/100%
Mohammed 2014 [24]	T1DM	66	14–18	16.0 ± 1.3	35/53.03%
Michaliszyn 2010 [25]	T1DM	16	12–17	14.4 ± 1.6	10/62.5%

**Table 3:** Key outcomes of CRF in patients with T1DM.

Study ID	Peak VO_2_ (L/min)	Peak VO_2_ (mL/kg/min)	Peak VE (L/min)	Peak Heart Rate (Beats/min)	HbA1 (%)	FBG (mg/dL)
Campaigne 1984 [13]	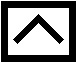	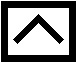	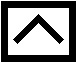	⊠	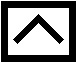	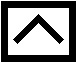
Faulkner 2010 [14]	⊠	⊠	N/A	N/A	⊠	N/A
Herbst 2007 [15]	N/A	N/A	N/A	N/A	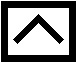	N/A
Mohammed 2021 [17]	N/A	N/A	N/A	N/A	⊠	⊠
Heyman 2007 [18]	N/A	N/A	N/A	N/A	N/A	N/A
Salem 2010 [19]	N/A	N/A	N/A	N/A	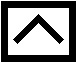	N/A
Shin 2014 [20]	N/A	⊠	N/A	N/A	⊠	N/A
Mohammed 2014 [21]	N/A	N/A	N/A	N/A	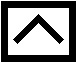	N/A
Michaliszyn 2010 [22]	N/A	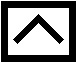	N/A	N/A	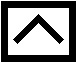	N/A

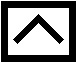
: significant improvement; ⊠: no significant difference; N/A: not applicable; FBG: fasting blood glucose; HbA1: hemoglobin A1.

**Table 4:** Key outcomes of lipid profile in patients with T1DM.

Study ID	Triglycerides (mg/dL)	Apolipoprotein B:A-1 Ratio	Cholesterol (mg/dL)	LDL Cholesterol (mg/dL)	HDL Cholesterol (mg/dL)	SBP (mmHg)	DBP (mmHg)
Herbst 2007 [15]	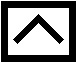	N/A	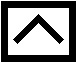	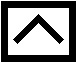	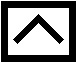	⊠	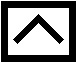
Mohammed 2021 [17]	⊠	N/A	⊠	⊠	⊠	⊠	⊠
Heyman 2007 [18]	⊠	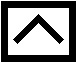	⊠	⊠	⊠	N/A	N/A
Salem 2010 [19]	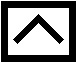	N/A	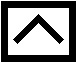	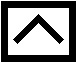	⊠	⊠	⊠
Shin 2014 [20]	⊠	N/A	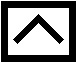	N/A	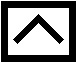	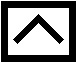	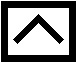
Mohammed 2014 [21]	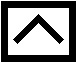	N/A	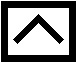	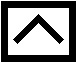	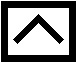	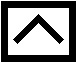	⊠
Michaliszyn 2010 [22]	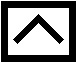	N/A	N/A	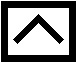	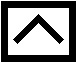	N/A	N/A

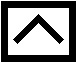
: significant improvement; ⊠: no significant difference; N/A: not applicable.

## Data Availability

Not applicable.

## Supplementary Materials

The following supporting information can be downloaded at: https://www.mdpi.com/article/10.3390/ijerph20021407/s1, Table S1. Preferred Reporting Items for Systematic reviews and Meta-Analyses extension for Scoping Reviews (PRISMA-ScR) Checklist.
